# Non-operative management for high-grade isolated renal trauma in pediatric patients: a case series

**DOI:** 10.11604/pamj.2023.44.71.36833

**Published:** 2023-02-06

**Authors:** Gede Wirya Diptanala Putra Duarsa, Paksi Satyagraha, Besut Daryanto

**Affiliations:** 1 Department of Urology, Faculty of Medicine, University of Brawijaya, Saiful Anwar General Hospital, Malang, East Java, Indonesia

**Keywords:** Kidney, pediatrics, hematuria, wounds, nonpenetrating

## Abstract

The kidney is the most commonly injured urinary tract organ in pediatric trauma with blunt mechanisms, causing around 80% of cases. Non-operative management (NOM) remained the first choice for minor blunt renal trauma; however, its value for major trauma is still under debate. We present three children with high-grade isolated renal trauma diagnosed using computed tomography scans and treated using NOM as the main strategy of treatment. The first patient (12-year-old) fully recovered without needing an auxiliary procedure. The second patient (6-year-old) developed urinoma and underwent percutaneous drainage of urinoma and double J stent (DJ) with an uneventful result. The third patient (14-year-old) developed urinoma and underwent percutaneous drainage and DJ stent. However, he experienced continuous hematuria that was treated via super-selective embolization. In conclusion, NOM for isolated high-grade renal trauma is feasible with good outcomes. If complications were developed during follow-up, minimally invasive procedures, such as super-selective angioembolization in continuing hemorrhage and initial drainage in urinoma, offered a comparable outcome without needing open surgery.

## Introduction

Abdominal trauma remains a significant cause of morbidity and mortality. The third most common abdominal organ involved in trauma is the kidney, after the spleen and liver [1]. Injury to the kidney occurs in 3.25% of trauma patients and involves 245.000 cases yearly, with blunt mechanisms of injury playing a major role [1,2].

Pediatric patients are more vulnerable to experiencing renal injuries due to their anatomic factors such as higher mobility, less perirenal fat, and their location [3,4]. American Association for the Surgery of Trauma (AAST) classification is a widely accepted tool for diagnosing patients with renal trauma. Non-operative management (NOM) is still the primary approach in managing patients with low-grade renal trauma (AAST I-II) and high-grade renal trauma (AAST III-V) with stable hemodynamic status due to the wider availability of computed tomography (CT) examinations in multiple centers and a better understanding of renal injury treatment [5]. Among those conditions, 95% of pediatric patients are relieved without surgery [1,3,4]. Renal trauma in one country may differ from that in another due to sociodemographic factors influencing trauma etiologies. Understanding the characteristics of renal trauma is useful in developing country-specific strategies or guidelines [6]. There have been very few reports of pediatric renal trauma in Indonesia. Here, we report three pediatric patients with isolated blunt high-grade renal trauma treated using NOM in our center.

## Methods

This is a case series of three pediatric patients presented with isolated blunt high-grade renal trauma and treated with NOM as the main strategy of treatment. This study was conducted in February 2022 at Saiful Anwar General Hospital Malang, East Java, Indonesia.

High-grade renal trauma was defined using the American Association for the Surgery of Trauma classification (AAST) as grades III-V [7]. At admission, all patients were resuscitated using intravenous 0.9% normal saline. They remained hemodynamically stable, with blood pressure maintained within a systolic 107 to 114 mm Hg range and a diastolic of between 59- and 77-mm Hg. NOM strategies include observation with supportive care, bed rest with vital signs, and laboratory test monitoring. If the patients devolved persistent hematuria or urinoma, they will be treated using minimally invasive procedures such as angioembolization or ureteral stenting [2]. The patients were followed up three months after the hospital admission. Clinical, laboratory, abdomen computed tomography (CT) scan, and retrograde pyelogram evaluations were taken to evaluate the successfulness of the procedure. The clinical characteristics, management, and outcomes of patients are summarized in Table 1.

**Table 1:** the brief description of the case series

No	Age (year)	Gender	Mechanism of trauma	Grad of trauma	Complications	Treatment
1	12	M	Fell from a tree	V	-	NOM
2	6	M	MVA	V	Large urinoma	NOM, percutaneous urinoma drainage, and DJ stent insertion
3	14	M	MVA	V	Hematoma with continuous hematuria	NOM, percutaneous drainage and DJ stent insertion, selective embolization

**Abbreviations:** M: male, MVA: motor vehicle accident, NOM: Non-operative management, DJ: double j.

**Ethical approval:** this study obtained an approval from The Ethics Commission of General Hospital Dr. Saiful Anwar Malang, East Java, Indonesia, with ethics ID: 400/044/CR/102.7/2022 on July 11^th^, 2022.

## Results

**Figure 1:**
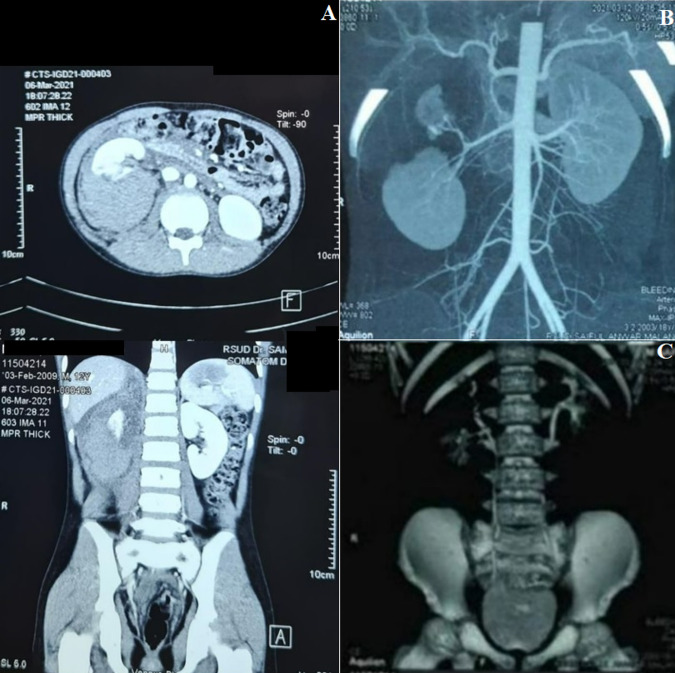
evaluation and clinical findings in the first patient A) initial CT scan showed viable upper, middle, and lower renal pole with intact contralateral kidney: coronal view (upper), axial view (lower); B, C) CT scan evaluation, intact vascular pedicle (upper), intact pelvicalyceal system (lower)

**Case 1:** a 12-year-old boy complained of right flank pain after falling from a three (3) meters with the primary impact in the right flank region and was diagnosed with suspected blunt trauma of the right kidney with stable hemodynamic status. Focused Assessment with Sonography for Trauma (FAST) showed a moderate free fluid collection in the retroperitoneal and perivesical regions. Intravenous contrast-enhanced abdominal computer tomography (CT) scan showed isolated grade V renal injury with discontinuity of upper renal pole and intact vascular pedicle, and large hematoma (Figure 1A). NOM was selected for his management approach. The patient was discharged after seven days in good condition. At three months of follow-up, the patient had a good clinical condition, and CT scan showed the resolve of renal damage without extravasation (Figure 1B).

**Figure 2:**
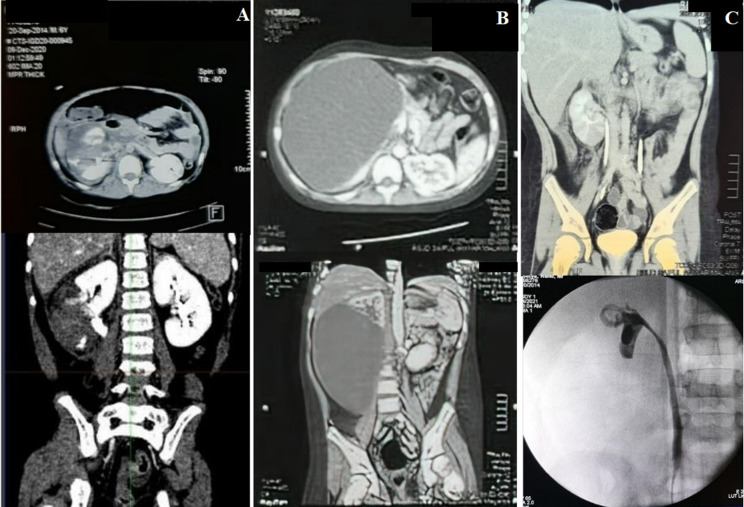
evaluation and clinical findings in the second patient A) initial CT scan showed right kidney laceration involving the pelvicalyceal system, segmental artery injury with leakage of urine in perirenal: coronal view (upper), axial view (lower); B) 1-week urgent CT scan evaluation, large urinoma in right kidney: coronal view (upper), axial view (lower); C) abdomen CT scan evaluation 1 month after to urinoma drainage (upper), RPG evaluation during DJ Stent removal (lower)

**Case 2:** a 6-year-old boy complained of right flank pain due to a motor vehicle accident (MVA) with primary impact in the right flank region, and patient access with multiple trauma rapid response with the stable hemodynamic status. Diagnostic confirmation using an abdomen CT scan with contrast was done (Figure 2A). The patient was diagnosed with high-grade renal trauma (AAST grade V) of the right kidney involving laceration into the pelvicalyceal system and segmental artery injury with leakage of urine in perirenal space. NOM was selected for his management approach. One week after the procedure, evaluation using a contrast-enhanced abdominal CT scan showed a large urinoma in the retroperitoneal space of the right kidney (Figure 2B). We performed percutaneous urinoma drainage, and DJ stent insertion on January 11^th^, 2021to the patient with an initial urinoma volume of 2000 cc was found, followed by administration of Ceftriaxone IV 2x1 gram and Nifedipine 3x2.5mg orally. The patient was discharged after ten days in good condition. At three months of follow-up, the patient had a good clinical condition, and the CT scan showed the resolve of renal damage without extravasation (Figure 2C).

**Figure 3:**
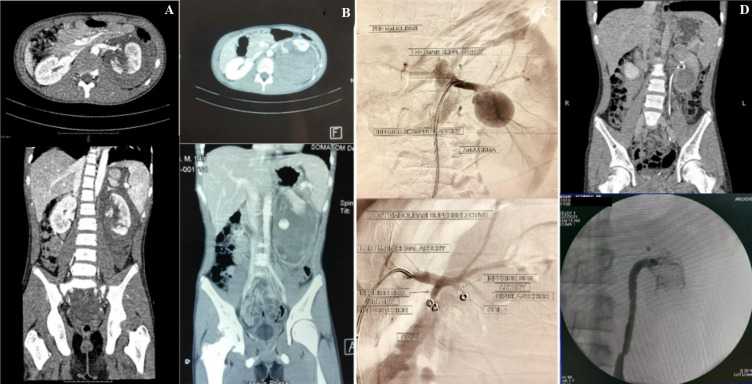
evaluation and clinical findings in the third patient A) initial CT scan showed laceration of left kidney with a form of shattered kidney: coronal view (upper), axial view (lower); B) 1-week urgent CT scan evaluation left kidney hematoma with a decrease of renal function secretion was found without any active bleeding and leakage: coronal view (upper), axial view (lower); C) super selective embolization; D) abdomen CT scan evaluation 1 month after drainage of hematoma and embolization (upper), RPG evaluation during DJ stent removal (Lower)

**Case 3:** a 14-year-old boy complained of left flank pain following MVA and was diagnosed with suspected blunt trauma of left renal with stable hemodynamic status. From the abdomen CT scan with contrast, we found a laceration of the left renal with formed of shattered renal and classified as high-grade renal trauma AAST grade V (Figure 3A), and NOM was performed as treatment.

One week after the procedure, evaluation using a contrast-enhanced abdominal CT scan showed a large urinoma in the retroperitoneal space (Figure 3B) that needed urinoma drainage and DJ stent insertion. However, he experienced continuous hematuria that was treated via super-selective embolization of the left kidney, followed by drug administration of Gentamicin IV 2x80 mg. Abdomen CT scan evaluation one month following drainage showed successful angiography procedure (Figure 3C). Follow-up three months later showed no sign extravasation and the result of CT images were normal (Figure 3D).

## Discussion

In this case series, we performed a NOM to manage high grade isolated blunt renal traumata in pediatric patients with successful outcome. The kidney is the third most commonly injured solid organ after blunt trauma and the second most commonly affected after penetrating trauma [8]. The treatment strategy for blunt renal trauma has not changed in the last 30 years. In most cases, the standard of care is NOM, and up to 95% of pediatric patients do not require surgery [9].

NOM is the treatment of choice for low grade (AAST grade 1-3) renal injuries and high grade (AAST grade 4-5) injuries with careful selection and hemodynamically stable pediatric patients [7]. Various peer-reviewed studies have been drawing the role NOM for hemodynamically stable pediatric patients with kidney injuries. For example, a study of 374 pediatric renal injuries demonstrated a >99% renal salvage rate [10]. They proposed that NOM of injuries Grades 1-3 necessitated a period of observation until hematuria resolved, at least 24 hours of bed rest for Grades 2 and 3, and a postinjury CT/functional scan at 3 months. For more severe injuries (≥ Grade 4), successful NOM required close observation, serial hematocrits, at least 48 hours of bedrest, and repeat imaging at 48 hours or earlier if clinically prompted to reassess the injury status [10]. Another series of 47 pediatric patients with high-grad isolated renal trauma was treated via NOM [11]. The authors demonstrate that implementation of a standardized NOM protocol was associated with significant improvements in care and decreased resource utilization in patients with isolated blunt renal injuries [11]. Our result is similar to mentioned studies. Despite the small number of patients in this study, significant improvements in care and resource utilization were achieved, which could be replicated by implementing a trauma-specific care protocol.

Despite the high success rate of NOM that reach up to 90% [11], in about 20% of renal trauma patients, significant complications may arise; for example, urinoma (1%) and post-trauma extravasation (2-18%) [2,12]. In general, most patients with urinoma or urine extravasation were successfully handled with conservative methods. Persistent urinomas should be treated percutaneously or endoscopically with ureteral DJ stent insertion, as performed in our second and third patients. This allows to produce a low-pressure system in the collecting system [13]. Persistent hematuria that does not resolve spontaneously from the bed rest method can also be treated with super-selective embolization, as performed in our third patient [14].

Nowadays, NOM is widely used to treat the majority of high-grade renal injuries since organ preservation is extremely desirable given the expected lifespan of patients [3]. In contrast, Alsaywid *et al*. mentioned that 28.5% of pediatric high-grade renal trauma patients required operative intervention [15]. Penetrating injuries such as gunshot and stab wound, multiple abdominal injuries, and hemodynamic instability were associated with the failure of NOM [16,17].

There is no definitive radiologic follow-up regimen after the blunt renal injury. Some authors recommend a urologic workup one year after trauma [18]. Some authors recommend reimaging within 24 to 36 hours of trauma for high-grade injuries because it may influence the timing and need for intervention [11]. In another study, the authors advised high-grade trauma patients with urinary extravasation to have an ultrasound or CT scan 2 to 4 weeks after discharge and blood pressure monitoring twice a year for 3 years [9]. We performed a follow-up radiologic image in all patients, showing the resolve of renal damage.

**Limitation:** this study has limited scope in terms of the age and body size encompassing pediatrics, a small number of participants, and isolated renal trauma as the main limitation.

## Conclusion

NOM showed good outcomes and remained the first treatment choice for isolated renal trauma with stable hemodynamic status, even in high-grade trauma. If complications were developed during evaluation, minimally invasive management offered a comparable outcome without the need for open surgery for renal function preservation.

### What is known about this topic

- *Non-operative management can be used as a primary approach in managing patients with high-grade renal trauma with stable hemodynamic status;*
- *Pediatrics are at greater risk of renal damage due to anatomical proportions, and their growing potential should be considered when picking treatments*.

### What this study adds

- *Non-operative management can be performed as a primary treatment in a pediatric patient with isolated high-grade hemodynamically stable renal trauma;*
- *The use of a standardized non-operative management protocol for pediatric patients suffering from isolated blunt renal injury improved outcomes and resource utilization;*
- *Complications can still occur during non-operative management, but these complications can be managed via minimally invasive management without the need for open surgery*.
